# Population Pharmacokinetic Analysis of Cefiderocol, a Parenteral Siderophore Cephalosporin, in Healthy Subjects, Subjects with Various Degrees of Renal Function, and Patients with Complicated Urinary Tract Infection or Acute Uncomplicated Pyelonephritis

**DOI:** 10.1128/AAC.01391-17

**Published:** 2018-01-25

**Authors:** Nao Kawaguchi, Takayuki Katsube, Roger Echols, Toshihiro Wajima

**Affiliations:** a Project Management Department, Shionogi & Co., Ltd., Osaka, Japan; b Infectious Disease Drug Development Consulting, LLC, Easton, Connecticut, USA

**Keywords:** acute uncomplicated pyelonephritis, augmented renal function, cefiderocol, cephalosporin, complicated urinary tract infection, pharmacokinetics, population pharmacokinetics, renal function, siderophore

## Abstract

Cefiderocol, a novel parenteral siderophore cephalosporin, exhibits potent efficacy against most Gram-negative bacteria, including carbapenem-resistant strains. The aim of this study was to perform a population pharmacokinetic (PK) analysis based on plasma cefiderocol concentrations in healthy subjects, subjects with various degrees of renal function, and patients with complicated urinary tract infection (cUTI) or acute uncomplicated pyelonephritis (AUP) caused by Gram-negative pathogens and to calculate the fraction of the time during the dosing interval where the free drug concentration in plasma exceeds the MIC (*fT*_MIC_). Population PK models were developed with three renal function markers, body surface area-adjusted estimated glomerular filtration rate (eGFR), absolute eGFR, and creatinine clearance, on the basis of 2,571 plasma concentrations from 91 subjects without infection and 238 patients with infection. The population PK models with each renal function marker adequately described the plasma cefiderocol concentrations. Clear relationships of total clearance (CL) to all renal function markers were observed. Body weight and disease status (with or without infection) were also significant covariates. The CL in patients with infection was 26% higher than that in subjects without infection. The *fT*_MIC_ values were more than 75% in all patients (and were 100% in most patients), suggesting that a sufficient exposure to cefiderocol was provided by the tested dose regimens (2 g every 8 h as the standard dose regimen) for the treatment of cUTI or AUP caused by Gram-negative pathogens.

## INTRODUCTION

Cefiderocol (product code S-649266) is a new injectable cephalosporin with a catechol group on the side chain at the C-3 position of the cephalosporin core that was initially identified by Shionogi & Co., Ltd., Osaka, Japan, and that exerts its antibacterial activity by inhibiting the synthesis of bacterial cell walls. Cefiderocol exhibits potent efficacy *in vitro* and *in vivo* against most Gram-negative bacteria, including carbapenem-resistant strains of Enterobacteriaceae, Pseudomonas aeruginosa, and Acinetobacter baumannii (1–4). Cefiderocol is being developed for the treatment of carbapenem-resistant Gram-negative bacterial infections, including nosocomial pneumonia, bloodstream infections, and complicated urinary tract infection (cUTI).

For cefiderocol, which exhibits bactericidal activity dependent on the duration of action, the pharmacokinetic (PK)/pharmacodynamic (PD) index most closely correlated with efficacy is the fraction of the time during the dosing interval where the free drug concentration in plasma exceeds the MIC (*fT*_MIC_) (5–7), as has been described with other cephalosporins (8, 9). *In vivo* animal infection models demonstrated a bacteriostatic effect at an *fT*_MIC_ of 40% to 70% and a bactericidal effect (≥1-log reduction) at an *fT*_MIC_ of 55% to 80% against carbapenem-resistant strains of Enterobacteriaceae, P. aeruginosa, and A. baumannii (5–7). Cefiderocol PK are linear over the range of 100 to 2,000 mg (10). Cefiderocol is mainly excreted unchanged via the kidneys (as 60% to 70% of the dose in subjects with normal renal function), and thus, the clearance of cefiderocol is dependent on renal function (11). The *in vitro* plasma protein binding of cefiderocol was 57.8%. The population PK model was previously developed on the basis of the concentration data of cefiderocol in healthy subjects and subjects with various degrees of renal function (12). The developed model well described the plasma concentration data.

The aim of this study was to perform a population PK analysis based on the plasma cefiderocol concentrations in healthy subjects, subjects with various degrees of renal function, and patients with cUTI or acute uncomplicated pyelonephritis (AUP) caused by Gram-negative pathogens in a phase 2 study of cefiderocol for the treatment of cUTI (13) and to calculate the *fT*_MIC_. A summary of the study designs is shown in Table S1 in the supplemental material. The population PK models were developed using three renal function markers: (i) the estimated glomerular filtration rate (eGFR), which was calculated by the modification of diet in renal disease (MDRD) equation (14) or an equation reported by Matsuo et al. for Japanese subjects (15) (the body surface area-adjusted eGFR [eGFRadj]); (ii) the eGFR converted by multiplying by the individual's body surface area and dividing by 1.73 m^2^ (absolute eGFR [eGFRabs]); and (iii) creatinine clearance (CL_CR_), which was calculated by the Cockcroft-Gault equation (16). These three renal function markers were assessed separately in the population PK analysis because they have been used as renal function markers (14, 17–19) and the selection of renal function markers might affect the prediction of cefiderocol PK. *fT*_MIC_ was calculated on the basis of simulated steady-state plasma cefiderocol concentrations and the MICs of Gram-negative uropathogens detected in the cUTI study.

## RESULTS

A total of 2,571 plasma cefiderocol concentrations obtained from 329 subjects were used for developing the population PK models. Data for samples stored under unstable conditions, samples with anomalous concentrations, or samples with concentrations below the limit of quantification (BLQ) (*n* = 264 concentrations) (see Materials and Methods) were excluded from the analysis.

The subject characteristics are shown in Table 1. The parameter estimates are provided in Table 2.

**TABLE 1:** Subject characteristics^*a*^

Characteristic	Value		
	Subjects without infection^*b*^ (*n* = 91)	Patients with cUTI or AUP (*n* = 238)	Overall
Body wt (kg)			
Mean (SD)	73.4 (17.3)	77.8 (16.1)	76.6 (16.5)
Median (range)	68.4 (45.1–124.1)	76.4 (46.3–138.0)	74.1 (45.1–138.0)
Age (yr)			
Mean (SD)	40.6 (15.7)	60.5 (16.3)	55.0 (18.4)
Median (range)	36.0 (20–74)	65.0 (18–93)	59.0 (18–93)
eGFRadj (ml/min/1.73 m^2^)			
Mean (SD)	86.3 (38.9)	70.8 (24.5)	75.1 (29.9)
Median (range)	99.0 (4–146)	72.0 (14–142)	77.0 (4–146)
eGFRabs (ml/min)			
Mean (SD)	89.8 (38.6)	77.2 (27.2)	80.6 (31.2)
Median (range)	99.0 (5–144)	78.0 (16–148)	83.0 (5–148)
CL_CR_ (ml/min)			
Mean (SD)	108.3 (48.2)	83.0 (31.9)	90.0 (38.7)
Median (range)	121.0 (7–185)	83.0 (25–186)	90.0 (7–186)
CL_CR_ (ml/min) for patients with cUTI (*n* = 175)			
Mean (SD)		81.3 (32.8)	
Median (range)		80.0 (25–186)	
CL_CR_ (ml/min) for patients with AUP (*n* = 63)			
Mean (SD)		87.6 (29.0)	
Median (range)		93.0 (32–159)	
Albumin concn (g/dl)			
Mean (SD)	4.2 (0.3)	4.1 (0.5)	4.1 (0.5)
Median (range)	4.2 (3.1–4.8)	4.2 (2.5–5.3)	4.2 (2.5–5.3)
Aspartate aminotransferase concn (U/liter)			
Mean (SD)	20.7 (8.1)	19.7 (11.5)	20.0 (10.7)
Median (range)	18.0 (10–45)	18.0 (6–101)	18.0 (6–101)
Alanine aminotransferase concn (U/liter)			
Mean (SD)	20.6 (10.4)	20.1 (16.6)	20.2 (15.1)
Median (range)	18.0 (5–51)	15.0 (4–111)	16.0 (4–111)
Total bilirubin concn (mg/dl)			
Mean (SD)	0.78 (0.38)	0.60 (0.30)	0.65 (0.34)
Median (range)	0.78 (0.20–2.00)	0.53 (0.19–2.88)	0.57 (0.19–2.88)
No. (%) of subjects by sex			
Male	75 (82.4)	108 (45.4)	183 (55.6)
Female	16 (17.6)	130 (54.6)	146 (44.4)
No. (%) of subjects by race			
White	23 (25.3)	230 (96.6)	253 (76.9)
Nonwhite	68 (74.7)	8 (3.4)	76 (23.1)
Asian	49 (53.9)	7 (2.9)	56 (17.0)
Black or African American	17 (18.7)	0 (0.0)	17 (5.2)
Native American or Alaska Native	1 (1.1)	0 (0.0)	1 (0.3)
Other	1 (1.1)	1 (0.4)	2 (0.6)

a CL_CR_, creatinine clearance calculated by the Cockcroft-Gault equation; eGFRadj, body surface area-adjusted estimated glomerular filtration rate; eGFRabs, absolute estimated glomerular filtration rate; cUTI, complicated urinary tract infection; AUP, acute uncomplicated pyelonephritis.

b Subjects without infection included healthy subjects and subjects with various degrees of renal function.

**TABLE 2:** Population PK parameter estimates for base model and final models^*e*^

Parameter	Units	Base model		Final model with eGFRadj^*a*^		Final model with eGFRabs^*b*^		Final model with CL_CR_^*c*^	
		Estimate	% RSE	Estimate	% RSE	Estimate	% RSE	Estimate	% RSE
OBJ		9,697.817		9,386.181		9,377.486		9,363.552	
PK parameters									
CL	liters/h	4.60	2.8	5.02	2.8	4.56	1.8	4.23	1.5
*V*_1_	liters	9.91	3.6	7.93	6.5	7.92	3.2	7.93	3.1
*Q*_2_	liters/h	5.81	7.2	5.81	22.0	5.78	5.6	5.75	5.3
*V*_2_	liters	5.37	3.8	5.41	4.4	5.41	3.4	5.41	3.3
*Q*_3_	liters/h	0.106	19.2	0.109	98.2	0.109	17.2	0.109	14.4
*V*_3_	liters	0.729	9.3	0.736	48.4	0.735	8.6	0.734	7.3
Effect of renal function marker^*d*^ on CL				0.631	12.4	0.621	3.5	0.653	3.9
Effect of body wt on CL				0.531	18.6				
Effect of body wt on *V*_1_				0.800	72.8	0.789	12.8	0.798	12.2
Effect of body wt on *V*_2_				0.689	26.4	0.673	10.8	0.698	17.3
Effect of disease status on CL						1.15	3.2	1.26	3.1
Effect of disease status on *V*_1_				1.35	5.1	1.36	5.1	1.36	4.9
% CV for IIV for CL (sh_ηp)		48.8 (1.4)	11.4	33.0 (2.9)	14.3	32.6 (2.9)	14.4	31.8 (3.1)	15.8
% CV for IIV for V1 (sh_ηp)		56.1 (8.8)	21.7	46.3 (11.1)	28.8	46.3 (11.0)	27.7	45.8 (11.1)	28.2
% CV for IIV for V2 (sh_ηp)		42.8 (32.2)	29.9	37.9 (34.2)	35.8	38.3 (34.2)	35.6	38.2 (34.2)	35.5
% CV for proportional residual error (sh_ε)		14.8 (14.9)	12.1	15.1 (14.2)	12.7	15.1 (14.2)	12.6	15.1 (14.1)	12.8

a CL = 5.02 · (eGFRadj/77.0)^0.631^ · (body weight/74.1)^0.531^; *V*_1_ = 7.93 · (body weight/74.1)^0.800^ · 1.35^disease status^ (disease status = 0 for subjects without infection and disease status = 1 for patients with infection; disease status is treated in the same way for the disease status superscripts in footnotes *b* and *c*); *V*_2_ = 5.41 · (body weight/74.1)^0.689^.

b CL = 4.56 · (eGFRabs/83.0)^0.621^ · 1.15^disease status^; *V*_1_ = 7.92 · (body weight/74.1)^0.789^ · 1.36^disease status^; *V*_2_ = 5.41 · (body weight/74.1)^0.673^.

c CL = 4.23 · (CL_CR_/90.0)^0.653^ · 1.26^disease status^; *V*_1_ = 7.93 · (body weight/74.1)^0.798^ · 1.36^disease status^; *V*_2_ = 5.41 · (body weight/74.1)^0.698^.

d eGFRadj, eGFRabs, or CL_CR_ for each model.

e CI, confidence interval; CL_CR_, creatinine clearance calculated by the Cockcroft-Gault equation; eGFRabs, absolute estimated glomerular filtration rate; eGFRadj, body surface area-adjusted estimated glomerular filtration rate; IIV, interindividual variability; sh_ηp, shrinkage in the standard deviation of interindividual variability parameters η; sh_ε, shrinkage in the standard deviation of intraindividual variability parameters ε; RSE, relative standard error.

A 3-compartment model was used as a structural PK model since the population mean parameters were estimated appropriately and were similar to those estimated in the previous population PK analyses (12). The proportional error model was selected, since the relative standard error (RSE; in percent) of the PK parameter estimates obtained using the combination error model were large (4.1% to 1,721%), which suggested that the model was not robust.

**FIG 1:**
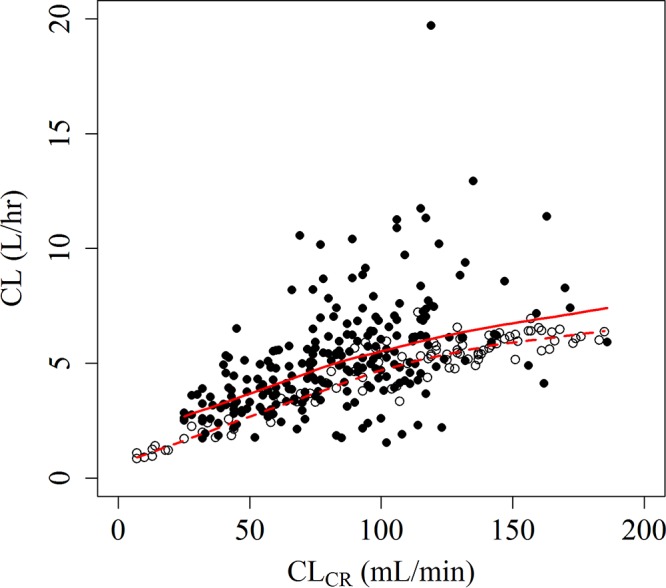
Relationship between CL and CL_CR_. Filled circles, patients with infection; open circles, subjects without infection; solid line, LOWESS (locally weighted scatterplot smoothing) line for patients with infection; dashed line, LOWESS line for subjects without infection.

The correlations of the renal function markers are presented in Fig. S1. The correlations for eGFRadj and eGFRabs were comparable, while they were slightly lower than the correlation for CL_CR_. Clear relationships of CL to eGFRadj, eGFRabs, and CL_CR_ were observed using the base model and are shown in Fig. 1 for CL_CR_ and Fig. S2 for eGFRadj and eGFRabs. Each renal function marker was a significant covariate for CL, and each was incorporated using the power model, which provided an objective function value (OBJ) similar to or less than that from the piecewise linear model and which had a smaller number of estimable parameters than the other model. The effects of body weight on CL and the volume of distribution in the central and peripheral compartments (*V*_1_ and *V*_2_, respectively) and the effect of disease status on *V*_1_ were significant in the final model with eGFRadj. The effects of body weight on *V*_1_ and *V*_2_ and the effect of disease status on CL and *V*_1_ were significant in the final models with eGFRabs and CL_CR_. The final model with CL_CR_ demonstrated that CL and *V*_1_ in patients with infection were 26% and 36% higher, respectively, than those in subjects without infection. The incorporation of significant covariates in each final model reduced the interindividual variability (IIV) for CL, *V*_1_, and *V*_2_ from that in the base model. In comparison with the IIV in the base model, in the final model with CL_CR_, IIV was reduced from 48.8% to 31.8% for CL, 56.1% to 45.8% for *V*_1_, and 42.8% to 38.2% for *V*_2_.

**FIG 2:**
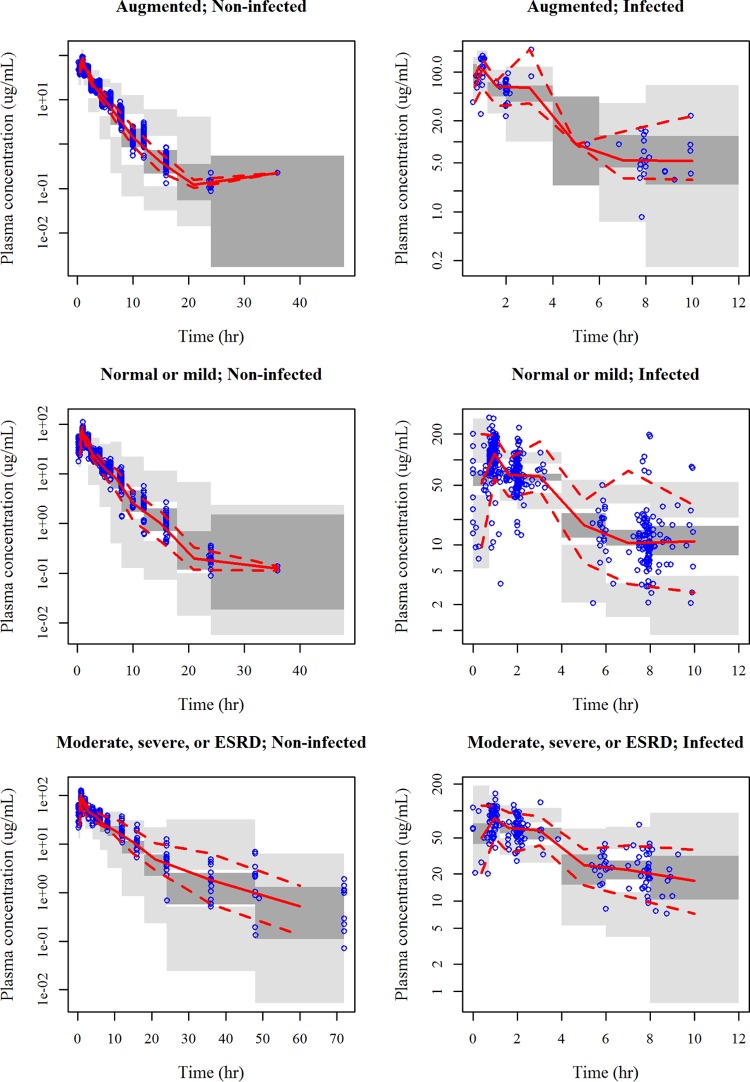
Visual predictive check for the final model with CL_CR_ by disease status and renal function group. The results for 200 simulations are shown, and data are presented on a semilogarithmic scale. Renal function groups defined by CL_CR_ were as follows: augmented renal function, CL_CR_ ≥ 120 ml/min; normal renal function or mild renal impairment, CL_CR_ = 60 to <120 ml/min; moderate or severe renal impairment or end-stage renal disease (ESRD), CL_CR_ = 5 to <60 ml/min. Noninfected, subjects without infection; Infected, patients with infection; Time, time after the previous dose; solid lines, observed median; dashed lines, observed 2.5th and 97.5th percentiles; dark gray shaded areas, model-predicted 95% confidence interval of the median; light gray shaded areas, model-predicted 95% confidence intervals of the 2.5th and 97.5th percentiles.

The number of estimable parameters was the same among the final models developed with eGFRadj, eGFRabs, and CL_CR_; and the typical parameter values, the IIV for each parameter, and the intraindividual variability were comparable among the final models (Table 2). Of the three final models, the OBJ of the model with CL_CR_ was the lowest, and thus, the calculation of *post hoc* PK parameters and *fT*_MIC_ was performed by using the final model with CL_CR_. The goodness-of-fit (GOF) plot for the final model with CL_CR_ is presented in Fig. S3. As shown in Fig. 2, the visual predictive check (VPC) indicated that the median predicted concentration profiles by disease status and renal function group well captured the observed data with a lack of bias. The prediction intervals in subjects without infection were relatively wide compared to the distribution of the observed data.

**FIG 3:**
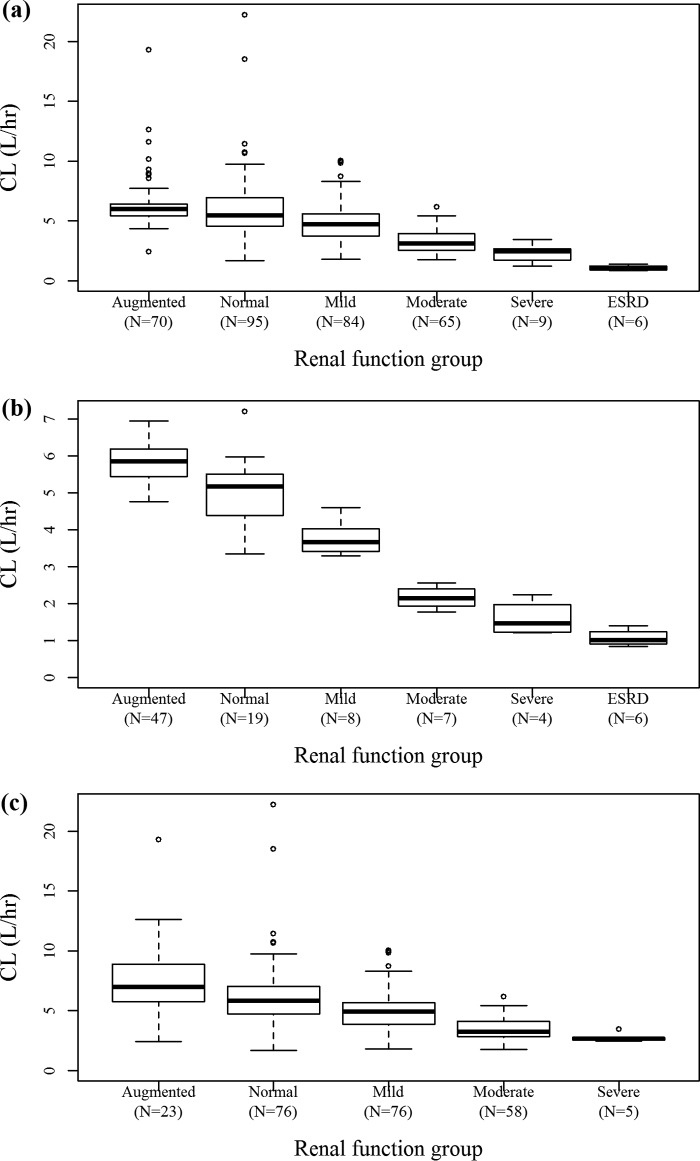
Box plots for individual *post hoc* CL values by renal function group defined by CL_CR_. (a) All subjects; (b) subjects without infection; (c) patients with infection. The final model with CL_CR_ was used to estimate individual parameters. Renal function groups defined by CL_CR_ were as follows: augmented renal function: CL_CR_ ≥ 120 ml/min; normal renal function, CL_CR_ = 90 to <120 ml/min; mild renal impairment, CL_CR_ = 60 to <90 ml/min; moderate renal impairment, CL_CR_ = 30 to <60 ml/min; severe renal impairment, CL_CR_ = 15 to <30 ml/min; end-stage renal disease (ESRD), CL_CR_ = 5 to <15 ml/min. Thick center lines, medians; top and bottom lines of the boxes, first and third quartiles (interquartile range), respectively; whiskers, the most extreme data within 1.5× the interquartile range; circles, outliers beyond 1.5× the interquartile range.

**FIG 4:**
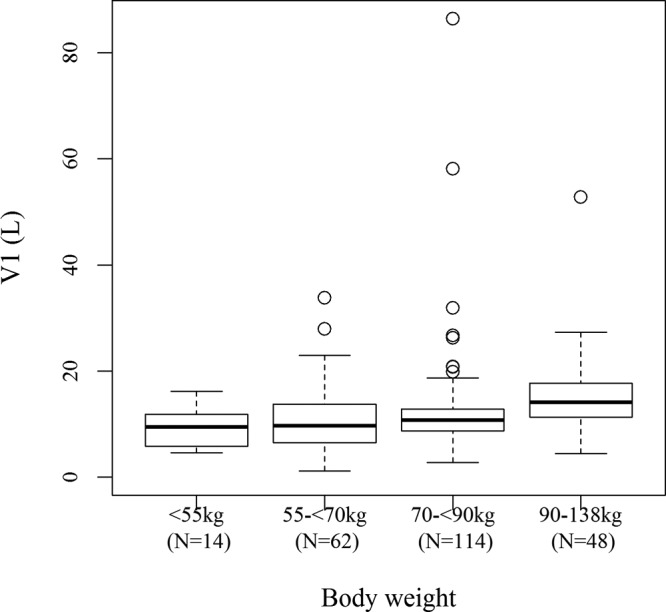
Box plot for individual *post hoc V*_1_ for patients with infection by body weight group defined by CL_CR_. The final model with CL_CR_ was used to estimate individual parameters. Thick center lines, medians; top and bottom lines of the boxes, first and third quartiles (interquartile range), respectively; whiskers, the most extreme data within 1.5× the interquartile range; circles, outliers beyond 1.5× the interquartile range.

Figure 3 shows box plots for individual *post hoc* CL values with empirical Bayesian estimation by renal function group (augmented and normal renal function; mild, moderate, and severe renal impairment; and end-stage renal disease [ESRD]). The CL of cefiderocol decreased with decreasing renal function. Figure 4 shows box plots for individual *post hoc V*_1_ values for patients with infection by body weight group (<55, 55 to <70, 70 to <90, or 90 to 138 kg). *V*_1_ was slightly dependent on body weight, which is consistent with the fact that body weight was a significant covariate on *V*_1_ in the population PK analysis. The maximum concentration (*C*_max_) and daily area under the concentration-time curve (AUC) for patients with infection, calculated using individual *post hoc* PK parameters, are summarized by dose regimen in Table 3. The daily AUC was similar among the dose groups.

**TABLE 3:** Summary of individual *post hoc* PK parameters for *C*_max_ and daily AUC for patients with infection^*a*^

Dose regimen	No. of patients	*C*_max_ (μg/ml)	Daily AUC (μg · h/ml)
2 g q8h	139	138 (29.5–460)	1,184 (270.0–3,562)
1.5 g q8h	26	134 (79.0–292)	1,186 (588.2–2,505)
1 g q8h	22	87.5 (57.0–161)	1,108 (588.4–1,719)
1.5 g q6h	8	102 (73.8–138)	862.0 (525.2–1,227)
1 g q6h	40	79.9 (30.7–122)	1,026 (316.4–1,686)
0.75 g q6h	3	69.3 (67.3–72.6)	1,003 (872.9–1,181)

a The values represent the mean (range). The final model with CL_CR_ was used to calculate the individual parameters for patients with infection. The dose regimen was tested in the phase 2 study of cefiderocol for the treatment of cUTI or AUP. q8h, every 8 h; q6h, every 6 h.

A summary of the MIC distribution for each pathogen is shown in Table S3. The calculated *fT*_MIC_ values based on the simulated steady-state plasma cefiderocol concentrations and the MIC of Gram-negative uropathogens detected in the cUTI study were more than 75% in all patients (and were 100% in most patients).

## DISCUSSION

We separately developed three population PK models based on plasma cefiderocol concentration data for subjects with or without infection by using different renal function markers (eGFRadj, eGFRabs, and CL_CR_). All models developed with the different renal function markers adequately described the plasma cefiderocol concentration data. These results suggest that any renal function marker could be used to adjust the cefiderocol dose.

eGFRadj and eGFRabs were similar, while they were slightly lower than that of CL_CR_ (see Fig. S1 in the supplemental material), which is consistent with well-known findings (14, 17). The MDRD equation is recognized as providing estimates of the GFR more accurate than those provided by the Cockcroft-Gault equation (18). On the other hand, there have been reports that the CL_CR_ estimated by the Cockcroft-Gault equation is closer to the measured creatinine clearance than the estimated GFR calculated with other equations, including the MDRD equation, for estimation of an augmented renal function (measured CL_CR_ > 130 ml/min) in critically ill patients (19). CL_CR_ was the best predictor of cefiderocol PK on the basis of the OBJ by the use of NONMEM software. However, the difference in the predictive performance among the models with each renal function marker would not be clinically significant. Therefore, it was suggested that any of these renal function markers can be used for dose adjustment and simulations based on renal function markers.

Body weight was a statistically significant covariate on CL, *V*_1_, and *V*_2_ in the final model with eGFRadj and *V*_1_ and *V*_2_ in the final models with eGFRabs and CL_CR_. Body weight was selected as a covariate on CL for the model with eGFRadj but not the models with eGFRabs and CL_CR_. This is probably because eGFRabs and CL_CR_ could accommodate the effect of body scale for describing the cefiderocol PK but eGFRadj could not. The *post hoc* analyses suggested that *V*_1_ was slightly dependent on body weight. However, the individual *V*_1_ values overlapped among the body weight groups, as shown in Fig. 4, and ratios of the median values of *V*_1_ relative to the typical value of *V*_1_ for infected patients (11.1 liters) were close to 1, with the values of the ratios being 0.85 for individuals weighing <55 kg, 0.87 for individuals weighing 55 to <70 kg, 0.96 for individuals weighing 70 to <90 kg, and 1.27 for individuals weighing ≥90 kg, suggesting that the effect of body weight on cefiderocol PK would not be clinically significant.

The disease status (with or without infection) was a significant covariate on *V*_1_ in the final model with eGFRadj and CL and *V*_1_ in the final models with eGFRabs and CL_CR_. The final model with CL_CR_ suggested that the values of CL and *V*_1_ in patients with infection were 26% and 36% higher, respectively, than those in subjects without infection. These results were consistent with the report for ceftolozane, a parenteral cephalosporin, in patients with cUTI (in which the values of both clearance and the volume of distribution were 21% higher in subjects with infection than subjects without infection) (20). The IIV for patients with infection was higher than that for subjects without infection, as shown in Fig. 2, which is probably because the plasma concentrations from the patients were limited (3 points per patient) and the IIV could not be calculated adequately.

The *fT*_MIC_ values were more than 75% in all patients (and were 100% in most patients), suggesting that the level of cefiderocol exposure obtained with the dose regimen used in the cUTI study (Table S2) would be sufficient for the treatment of cUTI and AUP caused by Gram-negative uropathogens (median MIC, 0.06 μg/ml; MIC range, 0.004 to 8 μg/ml; MIC_90_, 1 μg/ml). This sufficient exposure was expected from the results of Monte Carlo simulations, which indicated, using the PK model for healthy subjects, that a dose of 2 g every 8 h (q8h) with a 1-h infusion provided a high probability of attainment of a target of an *fT*_MIC_ of 75% against organisms with MICs up to 4 μg/ml (12). In addition, the mean urine cefiderocol concentrations for 8 patients in the cUTI study were 2,710 μg/ml (range, 953 to 5,520 μg/ml) at 2 h after the start of infusion and 1,520 μg/ml (range, 336 to 4,220 μg/ml) at 6 h after the start of infusion. Urine cefiderocol concentrations were also high relative to the MIC values detected in the cUTI study. As the protein-unbound fraction was not obtained in the phase 2 study of cefiderocol for the treatment of cUTI, individual *fT*_MIC_ values were calculated on the basis of the free concentrations in plasma using a fixed value for the unbound fraction of 0.422. The effect of the fixed unbound fraction would be minimal for the calculation of *fT*_MIC_ because the plasma unbound fraction was similar between the various renal function groups (11).

As shown in Fig. 3c, the clearance of cefiderocol in the 23 cUTI or AUP patients with augmented renal function (CL_CR_ ≥ 120 ml/min) was higher than that in the patients with normal renal function (CL_CR_ = 90 to <120 ml/min). Creatinine clearance was not measured in this study. Although the use of a measured creatinine clearance may be more appropriate to define augmented renal function, the use of an equation-derived value, such as CL_CR_ estimated by the Cockcroft-Gault equation, would be clinically practical. Monte Carlo simulations suggested that a more frequent dose (every 6 h) had a benefit for subjects with augmented renal function to attain a sufficient *fT*_MIC_ (12). The target patient population for cefiderocol includes critically ill patients infected with multidrug-resistant strains, which would be less susceptible than the uropathogens collected from the cUTI study. Augmented renal function is often observed in critically ill patients. Therefore, shortening of the cefiderocol dosing interval would be recommended for patients with augmented renal function to obtain enough exposure against organisms for which the MIC is higher.

In summary, our models developed with eGFRadj, eGFRabs, or CL_CR_ described well the PK of cefiderocol. Clear relationships of CL to renal function markers were observed, as expected. It was revealed that the exposure to cefiderocol in patients with infection would be modestly lower than that in subjects without infection. A cefiderocol exposure sufficient for the treatment of cUTI and AUP caused by Gram-negative uropathogens was provided by the tested dose regimens (2 g q8h as the standard dose regimen).

## MATERIALS AND METHODS

### Data.

Plasma cefiderocol concentration data from two phase 1 studies (10–12) and one phase 2 cUTI study (13) were used for the modeling (see Table S1 in the supplemental material). The population PK models were previously developed on the basis of plasma and urine cefiderocol concentration data for 54 healthy subjects and plasma concentration data for 37 subjects with various degrees of renal function (12).

In this study, the plasma concentration data for cUTI and AUP patients in the cUTI study (13) were included to develop the population PK models. The cUTI study was a multinational, double-blind, randomized study to assess the efficacy and safety of cefiderocol in hospitalized adults with cUTI with or without pyelonephritis or AUP caused by Gram-negative pathogens in comparison with intravenous imipenem-cilastatin (IMP-CS). The patients received 2 g as a 1-h intravenous infusion three times daily at 8-h intervals (q8h) for 7 or 14 days. The dose of cefiderocol was reduced on the basis of renal function and body weight, as shown in Table S2, consistent with the dosing instructions for IMP-CS, in order to maintain the blinding to the 2 treatments.

Blood samples for PK testing were not collected from 7 patients mainly due to patient withdrawal from the study. Blood samples for PK testing from 1 patient were not analyzed because the conditions used to store the sample did not meet the criteria required to maintain stability. Three concentrations from 1 patient were not used for analysis because the sampling times were unidentified. A total of 264 samples obtained after cefiderocol administration, which were mainly from healthy subjects, had concentrations that were BLQ and were excluded from the analyses. Plasma concentrations from 1 patient were entirely excluded from the analysis because they were all BLQ. A total of 156 blood samples from 52 patients were delayed in their delivery to the laboratory where the buffer was added to the samples for stabilization and had been stored at −20°C for more than 7 days after the samples were drawn but prior to the addition of the buffer; therefore, they were excluded from the analyses since cefiderocol is not thought to be stable in blood samples on the basis of stability data. Eight plasma concentrations obtained in the renal impairment study and the cUTI study were excluded from the analysis because they were considered anomalous, as the concentrations were much higher than the typical plasma concentrations, with the *C*_max_ being 153 μg/ml following a 2-g dose. After exclusion of these data, a total of 2,571 plasma cefiderocol concentrations from 329 subjects were used for the development of the population PK models. MIC data for 195 pathogens from 189 patients were used for calculation of the *fT*_MIC_. Subject characteristics obtained at the baseline were used (Table 1).

### Bioanalytical method.

The composite samples were prepared by treating plasma with a buffer (0.2 mol/liter ammonium acetate, pH 5) in a 1:1 ratio by volume and used for measurement of cefiderocol concentrations. The cefiderocol concentrations were determined using a validated high-performance liquid chromatography-tandem mass spectrometry (LC-MS/MS) assay. The assay was linear from 0.1 to 100 μg/ml for plasma. The precision and accuracy of the assay were 1.2% to 6.2% and −5.3% to 2.1%, respectively, for plasma. The lower limit of quantification of cefiderocol in plasma was 0.1 μg/ml.

### Population pharmacokinetic analyses.

A 3-compartment model was tested for describing the plasma concentration profiles of cefiderocol since the 3-compartment model well described the plasma cefiderocol concentration data for healthy subjects and subjects with various degrees of renal function (12). The 3-compartment model included the following parameters: CL, the volume of distribution in the central and peripheral compartments (*V*_1_, *V*_2_, and *V*_3_), and intercompartmental clearance (*Q*_2_ and *Q*_3_). The IIV for the PK parameters was assumed to follow a log-normal distribution and an exponential error model, defined as *P_i_* = TVP × exp(η_*P*,*i*_), where *P_i_* represents the value of the PK parameter for the *i*th individual, TVP represents the typical value of the population PK parameter, and η_*P*,*i*_ denotes the difference between the value of the PK parameter for the *i*th individual and the typical value of the PK parameter. IIV was considered for CL, *V*_1_, *Q*_2_, and *V*_2_. The proportional error model and the combination error model (an additive error model plus a proportional error model) were used to test for intraindividual (residual) variability.

After a base model was built, the influence of subject characteristics was assessed to build a covariate model. Renal function markers, body weight, age, sex, albumin (ALB) concentration, aspartate aminotransferase concentration, alanine aminotransferase concentration, bilirubin concentration, race, and disease status (subjects without infection or patients with infection) were tested as covariates on CL; and age, body weight, sex, race, ALB concentration, and disease status were tested as covariates on *V*_1_. Body weight was also tested as a covariate on *V*_2_, for which the IIV was estimable.

The effects of eGFRadj, eGFRabs, and CL_CR_ on CL were initially tested because clear relationships of CL to renal function markers were observed (Fig. 1 and S2), and the effects of the other covariates in the models with each renal function marker were tested. On the basis of visual inspection of the data (Fig. 1), the effect of the renal function markers on CL was tested by using a piecewise linear model and a power model. The cutoff value for the piecewise linear model was determined to be 100 ml/min/1.73 m^2^ or 100 ml/min for each renal function marker on the basis of data from a previous report (12).

Next, the effect of body weight on CL, *V*_1_, *Q*_2_, and *V*_2_ was tested by using the power model since body weight is generally a physiological factor that influences PK.

After incorporation of renal function markers and body weight, which showed significant effects, into the base model, the effects of the other covariates were tested using a screening with univariate addition. The continuous covariates were tested by using the power model, and the categorical covariates were tested by using a multiplicative model.

Covariates with a *P* value of <0.01 by the χ^2^ test and a change in the OBJ (ΔOBJ) of <−6.64 for 1 degree of freedom were included in the base model.

The covariates found to be significant as a result of the screening were included in the base model to construct a full model. To construct the final model, stepwise backward deletion was performed to delete from the full model the insignificant covariates, that is, those with a *P* value of <0.001 by the χ^2^ test and an ΔOBJ of <10.83 for 1 degree of freedom.

All population PK models were evaluated on the basis of GOF plots. A prediction-corrected VPC (21) was performed. In the binning for time, each bin was determined to correspond to each nominal sampling time.

### Calculation of *post hoc* PK parameters and *fT*_MIC_.

The calculation of *post hoc* PK parameters and *fT*_MIC_ was performed by using the final model with CL_CR_, which had the lowest OBJ of three final models with different renal function markers. *C*_max_ and the daily AUC for patients with infections were calculated by use of the final model with individual *post hoc* PK parameters with empirical Bayesian estimation and are summarized by dose regimen. The daily AUC was calculated as the daily dose divided by CL, whereas *fT*_MIC_ and *C*_max_ were calculated on the basis of steady-state plasma concentrations simulated every 0.25 h. The dose regimens of cefiderocol tested in the cUTI study, where the dose was adjusted on the basis of the patients' renal function and body weight (Table S2), were used in the summary (Table 3). Individual *fT*_MIC_ values were calculated on the basis of the free concentrations in plasma using a fixed value for the unbound fraction of 0.422 and the MIC of the Gram-negative uropathogen detected at the baseline.

### Software.

NONMEM (version 7.3.0) software (22) was used to estimate population PK parameters by the first-order conditional estimation with interaction and to execute simulations. Perl-speaks NONMEM (version 4.2.0) software (23) was used to execute the NONMEM run. R (version 3.0.3) software (24) was used to calculate individual *fT*_MIC_, *C*_max_, and AUC values on the basis of *post hoc* PK parameters and to create graphics.

## Supplementary Material

Supplemental material
