# Supplementary material for: Population Pharmacokinetic Analysis of Cefiderocol, a Parenteral Siderophore Cephalosporin, in Healthy Subjects, Subjects with Various Degrees of Renal Function, and Patients with Complicated Urinary Tract Infection or Acute Uncomplicated Pyelonephritis
Source: Antimicrob Agents Chemother. 2018 Jan 25;62(2):e01391-17. doi: 10.1128/AAC.01391-17 (PMC5786804; doi:10.1128/AAC.01391-17)
Supplement: Supplemental material [file supp_62_2_e01391-17__index.html]

Population Pharmacokinetic Analysis of Cefiderocol, a Parenteral Siderophore Cephalosporin, in Healthy Subjects, Subjects with Varying Renal Function, and Patients with Complicated Urinary Tract Infection or Acute Uncomplicated Pyelonephritis — Supplemental material 

# Population Pharmacokinetic Analysis of Cefiderocol, a Parenteral Siderophore Cephalosporin, in Healthy Subjects, Subjects with Various Degrees of Renal Function, and Patients with Complicated Urinary Tract Infection or Acute Uncomplicated Pyelonephritis

## Supplemental material

- Supplemental file 1 -

  Table S1, Table S2, Table S3, Figure S1, Figure S2, Figure S3

  PDF, 220K
