# Supplementary material for: Population Pharmacokinetic Analysis of Cefiderocol, a Parenteral Siderophore Cephalosporin, in Healthy Subjects, Subjects with Various Degrees of Renal Function, and Patients with Complicated Urinary Tract Infection or Acute Uncomplicated Pyelonephritis
Source: Antimicrob Agents Chemother. 2018 Jan 25;62(2):e01391-17. doi: 10.1128/AAC.01391-17 (PMC5786804; doi:10.1128/AAC.01391-17)
Supplement: Supplemental material [file AAC.01391-17_zac001186760s1.pdf]

Supplemental Materials

Table S1 Summary of Clinical Study Designs

**Study**	**Dose regimen**	**Subjects dosed cefiderocol**	**Plasma PK sampling**
Phase 1 single ascending single dose and multiple dose study for healthy subjects in Japan	Part 1:<br>Single cefiderocol 0.1-, 0.25-, 0.5-, 1-, 2-g doses or matching placebo infused over 1 hour<br><br>Part 2:<br>Multiple cefiderocol 1- or 2-g doses, or matching placebo infused over 1 hour q8hr for 10 days	Part 1:<br>30<br><br>Part 2:<br>24	Part 1:<br>Predose, 0.5, 1, 1.25, 1.5, 2, 2.5, 3, 3.5, 4, 4.5, 5, 6, 8, 10, 12, 16, 24, 36 and 48 hours from the start of the infusion<br><br>Part 2:<br>Day 1 (morning dose: first dose): predose, 0.5, 1, 1.25, 1.5, 2, 2.5, 3, 3.5, 4, 4.5, 5, 6, 8, 10, 12 and 16 hours<br>Days 2, 3, 5, 8 and 9 (each morning dose): predose<br>Day 10 (morning dose: last dose): predose, 0.5, 1, 1.25, 1.5, 2, 2.5, 3, 3.5, 4, 4.5, 5, 6, 8, 10, 12, 16, 24, 36 and 48 hours from the start of the infusion
Phase 1 renal impairment study in US	Single cefiderocol 1-g dose infused over 1 hour	38;<br>8 subjects each: normal renal function, mild and moderate renal impairment; 6 subjects: severe renal impairment; 8 subjects: ESRD requiring hemodialysis	Predose, 0.25, 0.5, 1, 1.5, 2, 3, 4, 5, 6, 8, 12, 16, 24, 36, 48 and 72 hours from the start of the infusion
Phase 2 study for patients with cUTI and AUP	Cefiderocol 2-g doses infused over 1 hour q8hr with adjustments for creatinine clearance and body size	300	Just prior to the infusion of the dose, −0.25 to 0 hours at the end of infusion, and 1 ± 0.5 hours after the end of infusion on Day 3

q8hr = every 8 hours

Table S2 Dose Regimen of Cefiderocol in the Phase 2 cUTI Study

Nominal Dose 6.0 g/day			
	**Creatinine Clearance (mL/min)**		
**Body Weight (kg)**	**≥ 71**	**41 – 70**	**21 - 40**
**≥70**	2.0 g q8hr	1.0 g q6hr	1.0 g q8hr
**60**	2.0 g q8hr	1.5 g q8hr	1.0 g q8hr
**50**	1.5 g q6hr	1.5 g q8hr	0.75 g q6hr
**40**	2.0 g q8hr	1.0 g q6hr	1.0 g q8hr
**30**	1.5 g q6hr	1.5 g q8hr	1.0 g q8hr

q8hr = every 8 hours, q6hr = every 6 hours

Table S3 MIC Distribution for Each Pathogen

	**MIC (μg/mL)**											
**Pathogen**	**<=0.004**	**0.008**	**0.015**	**0.03**	**0.06**	**0.12**	**0.25**	**0.5**	**1**	**2**	**4**	**8**
CITROBACTER FREUNDII	0	0	0	0	0	1	0	0	1	0	0	0
CITROBACTER FREUNDII COMPLEX	0	0	0	0	0	0	0	1	0	0	0	0
ENTEROBACTER CLOACAE	0	0	0	0	0	0	1	0	0	0	0	0
ENTEROBACTER CLOACAE COMPLEX	0	0	0	1	1	5	1	0	0	0	0	0
ESCHERICHIA COLI	13	3	8	22	21	20	12	8	6	4	1	0
KLEBSIELLA OXYTOCA	0	1	0	0	0	0	0	0	0	0	0	0
KLEBSIELLA PNEUMONIAE	0	3	4	1	3	2	9	4	4	3	3	1
MORGANELLA MORGANII	0	0	2	0	0	1	0	0	0	0	0	0
PROTEUS MIRABILIS	3	2	1	1	2	3	1	0	0	0	0	0
PSEUDOMONAS AERUGINOSA	0	1	0	3	2	2	2	0	0	0	0	0
SERRATIA MARCESCENS	0	0	1	0	0	0	0	0	0	0	0	0

MIC = minimum inhibitory concentration

Figure S1   Correlations of Renal Function Markers

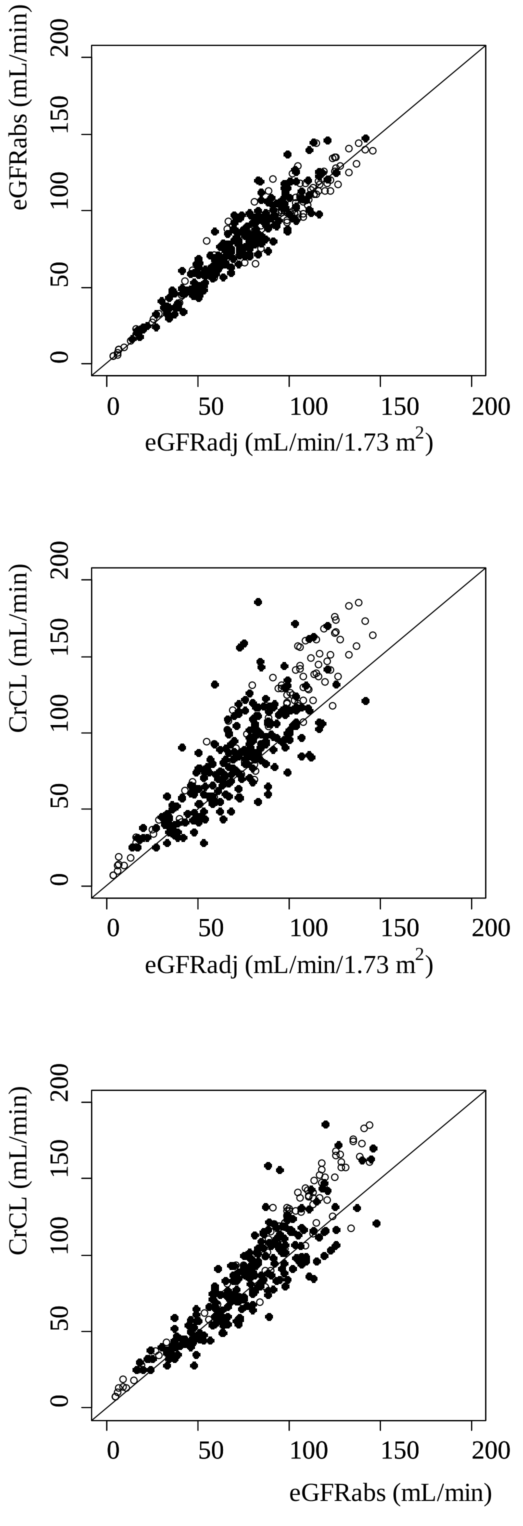

Filled circle: patients with infection. Unfilled circle: subjects without infection. Solid line: y = x.

Figure S2 Relationships between CL and eGFRadj and eGFRabs

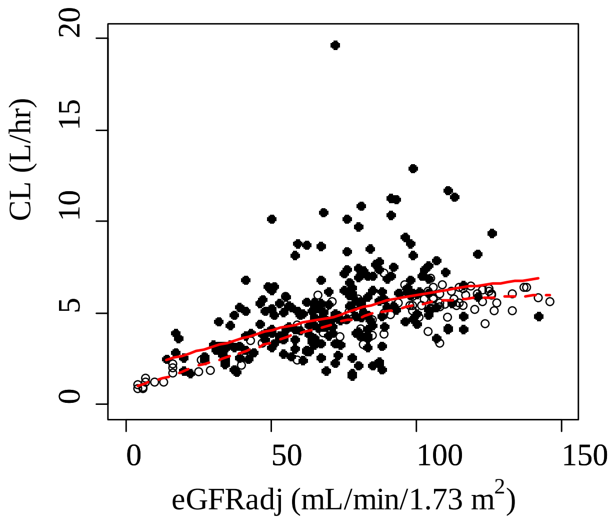

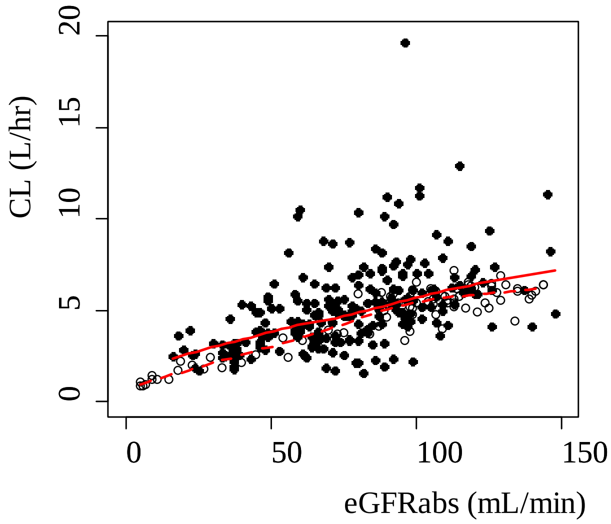

Filled circle: patients with infection. Unfilled circle: subjects without infection. Line: a LOWESS line (solid: patients with infection, dashed: subjects without infection).

Figure S3 Goodness-of-fit Plots for the Final Model with CrCL

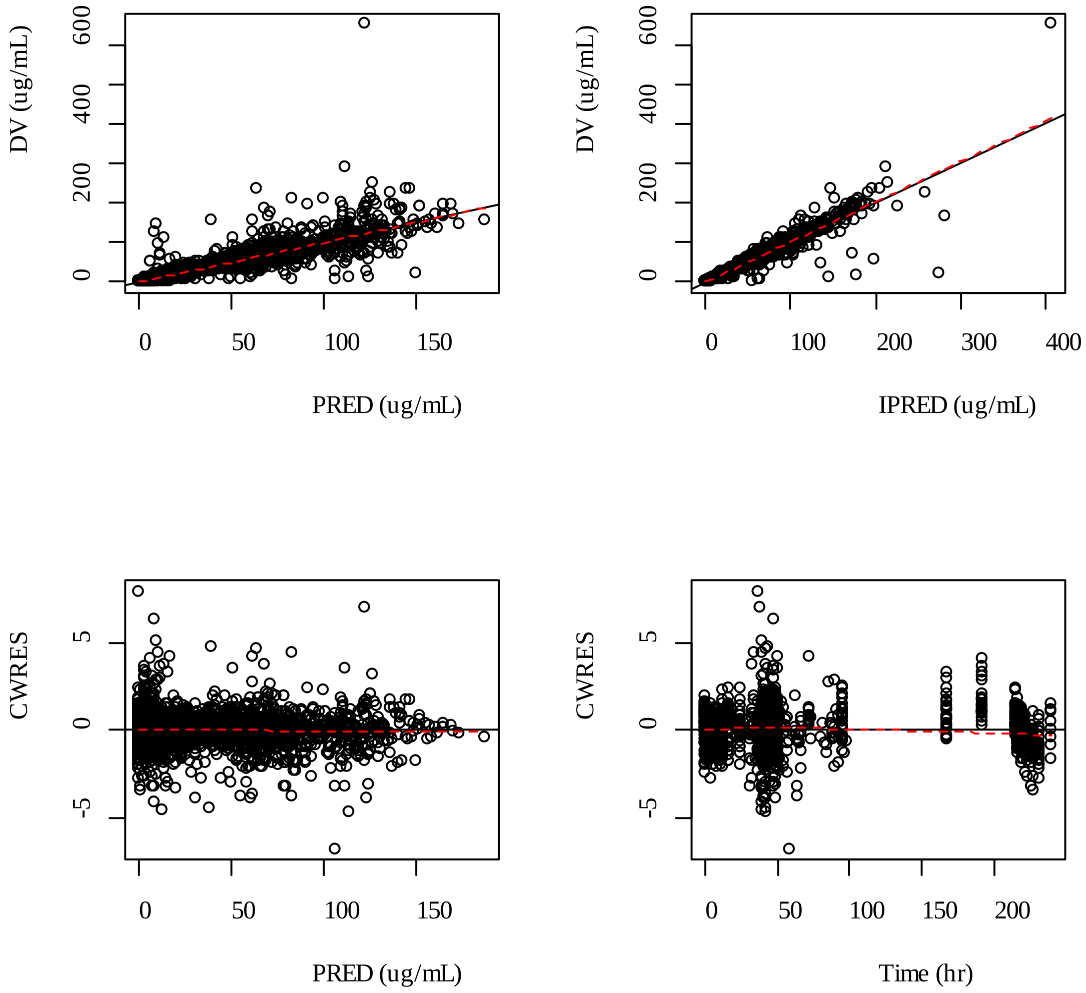

Solid line: y = x in upper figures and y = 0 in lower figures. Dashed line: a LOWESS line.
